# Molecular and Phylogenetic Evidence of Interfamilial Transmission of HTLV-1 in the Afro-Descendant Community of São José de Icatú in the Brazilian Amazon

**DOI:** 10.3390/v16081290

**Published:** 2024-08-13

**Authors:** Bruno José Sarmento Botelho, Wandrey Roberto dos Santos Brito, Gabriel dos Santos Pereira Neto, Janete Silvana Souza Gonçalves, Bruna Maria Silva Oliveira, Camille Marcela Camarinha de Oliveira, Aline Cecy Rocha de Lima, Sandra Souza Lima, Priscila de Nazaré Quaresma Pinheiro, Felipe Bonfim Freitas, João Farias Guerreiro, Ricardo Ishak, Antonio Carlos Rosário Vallinoto, Izaura M. Vieira Cayres Vallinoto

**Affiliations:** 1 Laboratory of Virology, Federal University of Pará, Belém 66075-110, Pará, Brazil; bruno.botelho@ics.ufpa.br (B.J.S.B.); wandrey.ben1@gmail.com (W.R.d.S.B.); gabrielnetoenf@gmail.com (G.d.S.P.N.); janetesilvanacosta@gamil.com (J.S.S.G.); bruna.silva.oliveira@ics.ufpa.br (B.M.S.O.); camille.oliveira@ics.ufpa.br (C.M.C.d.O.); alinececy@yahoo.com (A.C.R.d.L.); sandra.souza.lima@gmail.com (S.S.L.); rishak@ufpa.br (R.I.); 2 Postgraduate Program in Biology of Infectious and Parasitic Agents, Federal University of Pará, Belém 66075-110, Pará, Brazil; 3 Jurunas Basic Health Unit, Belém City Hall, Belém 66030-280, Pará, Brazil; priscilapcr4@gmail.com; 4 Retrovirus Laboratory, Evandro Chagas Institute, Anaindeua 67030-000, Pará, Brazil; felipebonfim@iec.gov.br; 5 Human and Medical Genetics Laboratory, Federal University of Pará, Belém 66075-110, Pará, Brazil; joaofg@ufpa.br

**Keywords:** HTLV-1/2, Amazon, quilombos, epidemiology, Afro-descendant community, vulnerable population

## Abstract

This study aimed to describe the prevalence of HTLV-1/2 in *quilombola* communities in the state of Pará and investigate the possible sociodemographic risk factors associated with the infection, as well as to trace the occurrence of the familial transmission of the virus. A total of 310 individuals living in eight *quilombos* located in the state of Pará (northern Brazil) were investigated for the presence of anti-HTLV-1/2 antibodies using an enzyme-linked immunosorbent assay (ELISA), and positive samples were confirmed using Western blot and/or real-time quantitative polymerase chain reaction (qPCR). Participants answered a questionnaire about sociodemographic aspects and risk factors for infection. Anti-HTLV-1/2 antibodies were detected in two individuals (one man and one woman), for an overall seroprevalence of 0.65%. Both individuals belonged to the community of São José de Icatú. The search for intrafamilial infection identified two other infected women, which increased the general prevalence of HTLV-1 among the Icatú to 6.25% (4/64). Western blot and qPCR confirmed their HTLV-1 infection, and phylogenetic analysis demonstrated that the isolates were of the cosmopolitan subtype and transcontinental subgroup. Epidemiological investigation of the cases revealed that the three women, at some point in their lives, had a relationship with the infected male individual. HTLV-1 is transmitted silently between individuals in the community of São José de Icatú with a present or past family relationship, stressing the need for screening and laboratory diagnosis to prevent further dissemination of the virus and surveillance of disease emergence.

## 1. Introduction

In 1980, type C retrovirus particles were identified in a patient with cutaneous T-cell lymphoma, the agent being called human T-cell leukemia virus 1 (HTLV-1) [1]. Two years later, from an individual carrying a rare type of hairy cell leukemia, another retrovirus related to HTLV-1, named HTLV-2, was isolated from an individual harboring a rare type of hairy cell leukemia [2]. More recently, two new viral types, HTLV-3 and HTLV-4, were described in hunting populations of nonhuman primates living in isolated forests in Cameroon, central Africa [3].

These viruses are members of the *Retroviridae* family, *Orthoretrovirinae* subfamily, and *Deltaretrovirus* genus [4]. They are transmitted by sexual contact [5,6,7], by sharing syringes/needles [8], through the transfusion of contaminated blood [9], through organ transplants [10], and vertically from infected mothers to their children, mainly through long-term breastfeeding [11,12,13,14]. Sexual transmission and breastfeeding impact the occurrence of intrafamilial transmission of HTLV-1/2, as described in several studies from the following locations: Argentina [15,16]; China [17]; Brazil, especially in Japanese immigrants and their descendants [18,19]; in the city of Salvador, Bahia [20]; in blood donors in Northeast Brazil [21]; in an isolated Afro-Brazilian community in Mato Grosso do Sul [22]; and in people who use illicit drugs in the state of Pará [23].

HTLV-1 has a heterogeneous distribution worldwide and is endemic in some regions, such as the Caribbean, Japan, Australia–Melanesia, Central Africa, and South America [24]. HTLV-2, on the other hand, is hyperendemic in indigenous populations in South America [7,13,25]. In Brazil, it is estimated that approximately 2.5 million people are infected with HTLV-1/2, making it the country with the most cases in the world [26,27,28]. The states of Bahia, Maranhão, and Pará have the highest prevalence of HTLV-1 [26,28,29,30], and its occurrence is attributed to ancient human migratory flows [31].

The state of Pará has high endemicity for HTLV-2 in indigenous peoples [7,13,32], and both viral types (HTLV-1/2) are found in urban locations, riverside populations, and Afro-descendant communities called *quilombos* [33,34,35,36]. The distribution of the virus in *quilombola* territories is not well known, given the large number of communities present in the state (a total of 516) and the low number of communities studied [37].

*Quilombola* communities were formed during the colonial period by enslaved African individuals who fled plantation farms in search of freedom, becoming autonomous communities with their own historical trajectory [38]. The presence of HTLV-1/2 in these communities has been attributed to the African origin of their inhabitants [31,34]. Many of the remaining *quilombo* communities suffer from a lack of access to quality public healthcare and from exposure to communicable diseases, making them vulnerable to various health problems [39,40].

In this study, we aimed to describe the prevalence of HTLV-1/2 in quilombola communities in the state of Pará and investigate the possible sociodemographic risk factors associated with the infection, as well as to trace the occurrence of familial transmission of the virus.

## 2. Materials and Methods

### 2.1. Study Population and Sample Collection

**Figure 1:**
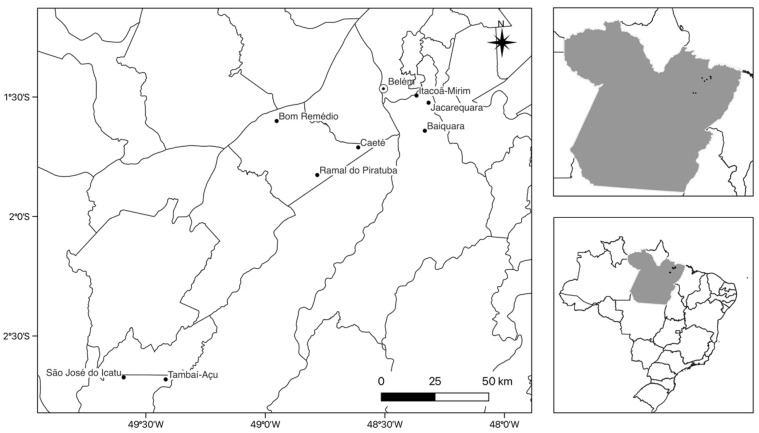
Geographic location of the *quilombola* communities investigated for the presence of HTLV-1/2 infection, located in the state of Pará (Brazil).

Between the months of February 2022 and November 2022, seven quilombo communities were visited to collect sociodemographic and behavioral information on the risk factors for HTLV-1/2 infection. A total of 310 individuals living in the communities of Itacoã-Mirim (n = 31), Jacarequara (n = 34), and Baiaquara (n = 13), located in the municipality of Acará, Bom Remédio (n = 34), Caeté (n = 13), and Ramal do Piratuba (n = 26), municipality of Abaetetuba, and São José do Icatú (n = 64) and Tambaiaçú (n = 95), municipality of Mocajuba (Figure 1), participated in the study.

Individuals who agreed to participate in the study answered a questionnaire about the sociodemographic aspects and risk factors for infection. Similarly, all of the blood samples (4 mL) were collected using venipuncture, separated into plasma and leukocytes after centrifugation (5000 rpm/5 min), and stored at −20 °C until laboratory analysis.

### 2.2. Ethical Aspects

This study was approved by the National Research Ethics Commission, CONEP (CAAE: 27290619.2.0000.0018), in compliance with Resolution No. 466/12 of the Ministry of Health, which regulates research involving human beings. All individuals who agreed to participate in the study signed a consent form. In cases where individuals were illiterate, consent was obtained from a legal representative of the family.

### 2.3. Serological Analysis

An enzyme-linked immunosorbent assay (ELISA) (Murex HTLV-I+II, DiaSorin, Dartford, UK) was used for the detection of anti-HTLV-1/2 antibodies in the plasma according to the manufacturer’s instructions. Those whose results were reactive or indeterminate had their plasma subjected to Western blotting (HTLV BLOT 2.4 DiaSorin) to confirm and differentiate between HTLV-1 and HTLV-2 infection.

### 2.4. DNA Extraction

Molecular confirmation of infection and differentiation of the viral type were performed using a multiplex real-time PCR. DNA was extracted from the seropositive samples using the QiaAmp DNA Mini Kit (Qiagen, Hilden, Germany) following the manufacturer’s protocol.

### 2.5. Real-Time PCR

Real-time PCR was performed using the TaqMan^®^ Universal Master Mix (Applied Biosystems, Foster City, CA, USA) with three target sequences, the albumin gene (141 bp) as an endogenous control, the pol gene (186 bp) from HTLV-1, and the tax-2 gene (75 bp) from HTLV-2 [41]. The sequences of the primers used were 5′-CCCTACAATCCAACCAGCTCAG-3′ (HTLV-1F), 5′-GTGGTGAAGCTGCCATCGGGTTTT-3′ (HTLV-1R), 5′-CGATTGTGTACAGGCCGATTG-3′ (HTLV-2F), 5′-CAGGAGGGCATGTCGATGTAG-3′ (HTLV-2R), 5′-GCTGTCATCTCTTGTGGGCTGT-3′ (Albumin F), and 5′-AAACTCATGGGAGCTGCTGGTT-3′ (Albumin R); the probe sequences used were JOE-5′-CTTTACTGACAAACCCGACCTACCCATGGA-3′-BHQ (HTLV-1), FAM-5′- TGTCCCGTCTCAGGTGGTCTATGTTCCA-3′-MGB (HTLV-2) and NED-5′-CCTGTCATGCCCACACAAATCTC-3′-MGB (albumin). qPCR was performed with the following mix: 12.5 μL of Master Mix, 6.0 μL of water, 1.0 μL of assay-by-design (primer and probe set), and 0.5 μL of DNA in a final volume of 20 μL. The cycling protocol was as follows: one cycle of 50 °C for 2 min; a cycle of 95 °C for 10 min; 50 cycles of 90 °C for 50 s; and a final cycle of 60 °C for 1 min.

### 2.6. Nested PCR

The samples confirmed to be positive for HTLV-1 using qPCR were subjected to molecular subtype identification through nested PCR of the 5′ LTR-1 region (788 bp). For the first reaction, 15.95 μL of ultrapure water (H_2_O), 1.25 μL of buffer (10×) 1.5 μL of MgCl_2_ (50 mM), 3.0 μL of dNTPs (10 mM), 0.5 μL of each primer (20 pmol), 0.3 μL of Taq DNA polymerase (1 U/μL), and 2.0 μL of DNA were combined. The following HTLV-1 primer sequences were used: LTR-I.01, 5′-TGACAATGACCATGAGCCCCAA-3′, and LTR-I.02, 5′-CGCGGAATAGGGCTAGCGCT-3′. The second reaction followed the same protocol using the primers LTR-I.03 (5′-GGCTTAGAGCCTCCCAGTGA-3′) and LTR-I.04 (5′-GCTAGGGAATAAAGGGGCGC-3′) and 2.0 μL of the PCR from the first reaction. Both reactions employed the same temperature cycle: 94 °C for 5 min; 35 cycles at 94 °C for 30 s, 61 °C for 30 s, and 72 °C for 40 s; and finally, 72 °C for 10 min.

### 2.7. DNA Sequencing and Phylogenetic Analysis

After purification of the PCR product (5′LTR region), the amplified DNA was sequenced using the Sanger method with a BigDye Terminator v3.1 cycle sequencing kit (Thermo Fisher, Waltham, MA, USA) using a Genetic Analyzer 3130xl (Applied Biosystems, Woburn, MA, USA) [42].

The sequences obtained were analyzed using the Chromas program 2.6.6v (Technelysium—DNA Sequencing Software). The quality of each sequence was analyzed with the Phred algorithm, defined as a base value above 30, which represents 99.9% sequencing accuracy. The assembly of contigs (a combination of sense and antisense sequences) was carried out using BioEdit software 7.2.5v (Biological Sequence Alignment Editor). LTR sequence alignment was performed using Geneious Prime software 2024.0.2v (www.genious.com, Biomatters, Auckland, New Zealand, accessed on 20 January 2024).

Phylogenetic analysis was performed using reference strain sequences published in GenBank representing HTLV-1 subtypes with worldwide distributions, including strains from different regions of Brazil. A maximum likelihood (ML) tree was constructed in MEGA 11.0.13v [43] using initial neighbor joining (NJ) trees and the Tamura-Nei (TN) model, including substitutions, transitions, transversions, gamma distribution rates between sites with a gamma parameter, homogeneous patterns between lineages, and partial deletions with a 95% cutoff as initial trees. ML trees were constructed using TN G+I with five discrete gamma categories and gaps were treated with partial deletion at a 95% cutoff. The heuristic method included extensive pruning of subtrees and moderate branch grafting. To infer the bootstrap values, 1000 bootstrap-to-tree interactions were performed. Layout changes were made in FigTree 1.4.4v (http://tree.bio.ed.ac.uk/software/figtree/; accessed on 20 January 2024). Common polymorphisms were analyzed by aligning the samples with a reference strain using BioEdit software via the ClustalW multiple-sequence alignment algorithm.

The strains used in the phylogeny and their access codes in GenBank were as follows: ICA#39 (PP757780); ICA#76 (PP757779); ICA#83 (PP757778); FNN100 (DQ005547.1); TSP12705 A (DQ897684.1); FNN153 A (DQ005550.1); BBD 3110 (FJ911637.1) BBD 2420 (FJ911628.1) TSP12481 A (DQ897683.1); SNT92 A (DQ070892.1); SNT43 A (DQ070891.1); Me2 Peru (Y16479.2); Qu1 Peru (Y16475.1); 2472LE A (AY818430.1); AINU (D23694.1); BOI (L36905.1); ATK (J02029.1); H5 (M37299.1); JPNBr177 (AY499187.1); TA6 (U53074.1); TA7 (U53075.1); 0D (U12805.1); BD89112 S (DQ235698.1); Bo (U12804.1); GH78 (D23693.1); PH906 C (AY342303.1); PH907 C (AY342304.1); 2656ND G (AY818431.1); H23 (L76312.1); 1259NG (AY818424.1); 1127MO B (AY818433.1); 1842LE D (AY818429.1); 2810YI G (AY818432.1); 979MO B (Y818423.1); StDen (L76306.1); 1380MV B (AY818425.1); рн236А (L76307.1) (1443MV B AY818426.1); Lib2 (Y17017.1); 1503MV B (AY818427.1); Lib1 (Y17016.1); Efe1 (Y17014.1); and Mel5 (L02534.1).

### 2.8. Statistical Analysis

The collected information was stored in the Epiinfo 7.2 database. Sociodemographic characteristics and risk factors were presented through descriptive statistics using the R 4.2.2 program.

## 3. Results

### 3.1. Prevalence of Infection

Anti-HTLV-1/2 antibodies were detected in 2 of the 310 individuals studied, for an overall seroprevalence of 0.64% (95% CI: 0.1–2.6%). Both individuals were from the community of São José do Icatú, for a prevalence of 3.12% (2/64) in this population (95% CI: 0.5–11.8%). Western blot analysis confirmed HTLV-1 infection in two individuals. qPCR amplification was confirmed in only one individual (Table 1).

### 3.2. Index Cases

The first patient was a black woman who was 54 years old (ICA #005), a widow, with three past relationships and a current one with the case ICA #039 (described below), had incomplete primary education, and had an income less than the minimum wage. She had no tattoos or piercings, did not use illicit drugs, and had never received a blood transfusion. She reported having been breastfed as a child, being sexually active, having had seven pregnancies, and having breastfed her children for 6 months or more. She reported having had her first sexual intercourse at age 18, not using condoms during sexual intercourse, having one sexual partner per week, and not having had a previous diagnosis of a sexually transmitted infection (STI).

The second case involved a 69-year-old black man (ICA #039) in a relationship with four women in the community, among them being the index case ICA #005, with an incomplete primary education and an income less than the minimum wage. He had no tattoos or piercings and did not use illicit drugs. He reported having received a blood transfusion in 2017, being sexually active, having had his first sexual intercourse at age 15, having had sex in exchange for money, and not having been diagnosed with an STI. He also stated that he had two sexual partners a week and sometimes used condoms.

### 3.3. Family Screening

**Figure 2:**
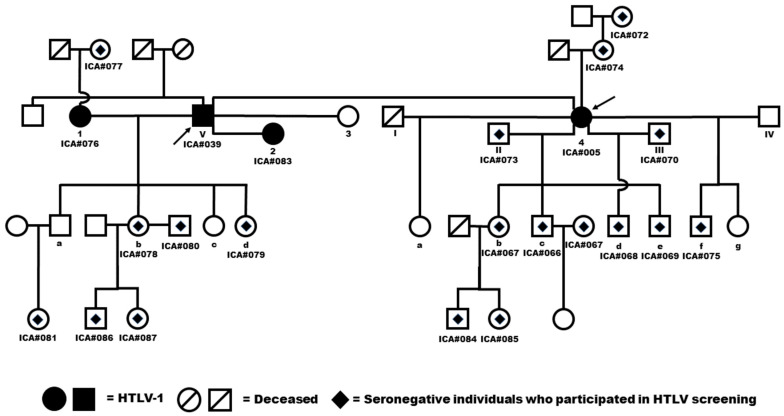
Pedigree of the investigated family members of the index cases from Icatú. This Figure demonstrates a complex and unusual case of sexual relationships between HTLV-positive individuals in the community. In the first serological screening, we identified two HTLV-1 positive individuals, a woman (ICA #005) and a man (ICA #039), who are highlighted by arrows. An interview with these individuals revealed a previous sexual relationship between them, and it is possible to see that the man (ICA #039) also had sexual relationships with the other two HTLV-1 positive women (ICA #076 and ICA #083). Indo-Arabic numerals: Horizontal relationships in chronological order of the index case. Roman numerals: Horizontal relations in chronological order of the first confirmed case. Lower letters: Birth of the children in chronological order.

The search for family members of the two index cases of confirmed HTLV-1 infection in the Icatú community identified 21 other family members whom we contacted and tested for the infection. Two new individuals, the only ones who still reside in the community, were positive for HTLV-1 (9.52%; 2/21), both of whom were women who were former partners of the man aged 69 described above (Figure 2). The findings of these two infected women increased the general prevalence of HTLV-1 among the Icatú to 6.25% (4/64).

The first of these two was a 54-year-old black woman (ICA #076) with an incomplete primary education and a family income less than the minimum wage. She had no tattoos or piercings, had not received a blood transfusion, did not use illicit drugs, was sexually active, was breastfed as a child, had four pregnancies, and breastfed her children for 6 or more months. She reported that the age of her first sexual intercourse was 16 years, she did not have sex in exchange for money, she did not use condoms, and she was not diagnosed with an STI.

The second was a 66-year-old black woman (ICA #083) with an incomplete primary education and a family income less than the minimum wage. She did not have tattoos or piercings, had not received a blood transfusion, and did not use illicit drugs. She was no longer sexually active, as a child she was breastfed, she had had ten pregnancies, and she breastfed her children for 6 or more months. Her first sexual intercourse was 13 years old, she did not have sex in exchange for money, she did not use condoms, and she was not diagnosed with an STI.

The investigation of possible interrelationships between the four positive individuals revealed the existence of sexual relations in the past and present between the man and the three women. 

### 3.4. Sociodemographic Profile and Associated Risk Factors

Among the 310 investigated individuals (Table 2), the majority were women (74.8%). The average age was 38.9 years, and the median age was 36 years (Q1: 23; Q3: 54). Most of the participating individuals declared themselves black (73.9%), with only literacy (49.7%—two to three years of study) as their last completed level of education, and also reported having an income of less than the minimum wage (69.7%). HTLV-1-infected individuals presented a similar profile.

When considering the possible risk factors (Table 3), such as infected individuals, the majority said they did not have a tattoo (86.1%), had no piercings (97.7%), and did not use illicit drugs (93.9%). The majority were breastfed as children (94.5%), were sexually active (70%), did not use condoms during sexual intercourse (43.9%), and had one partner per week (70.3%). The average age at first sexual intercourse was 16.5 years (SD = 3.22), and the median was 16 years (Q1: 15; Q3:18).

### 3.5. Phylogenetic Analysis

**Figure 3:**
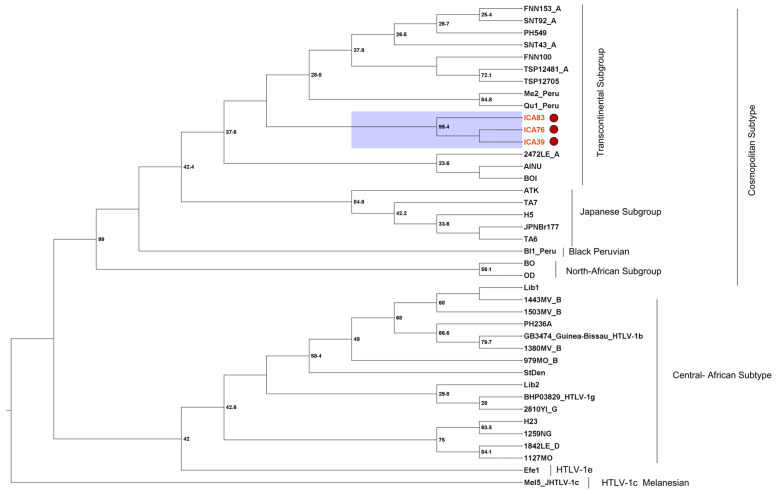
Maximum likelihood phylogenetic analysis using 40 HTLV-1 LTR sequences including the samples ICA #039, ICA #076, and ICA #083. The Mel5 and Efe1 sequences were used as outgroups. The tree with the highest log likelihood (−1041.33) is shown. The percentage of trees in which the associated taxa clustered is shown next to the branches. The initial tree(s) for the heuristic search were obtained by applying the neighbor-joining method to a matrix of pairwise distances estimated using the Tamura–Nei model. A discrete gamma distribution was used to model differences in evolutionary rate between sites (five categories (+G), parameter = 0.5886). The rate variation model allowed some sites to be evolutionarily in-variant ([+I], 0.00% of sites). The statistical support was applied using 1000 bootstrap replicates. Gen-bank codes: ICA#39 (PP757780); ICA#76 (PP757779) and ICA#83 (PP757778).

The amplification of the 5′ LTR region in three of the infected individuals showed that they formed a monophyletic clade with a bootstrap of 99.4% in the cosmopolitan subtype (HTLV-1a) and transcontinental subgroup (A) (Figure 3). The three samples presented the same polymorphisms in the nucleotide sequence of the 5′ LTR region (Table 4), and presented a nucleotide similarity of 99.6% when compared to each other and an average of 97.4% in relation to the other samples of the Transcontinental sub-group.

## 4. Discussion

The continuous and active search for new cases of infection in the remaining *quilombo* communities in the state of Pará led us to the first report of HTLV-1 infection in the São José do Icatú community in the municipality of Mocajuba, Pará, which confirms that *quilombos* are population groups with a high chance of infection. The prevalence found in São José do Icatú is much greater than that described in other *quilombola* communities in the same state, which have ranged from 0 to 2.06% [33,34], as well as the prevalence of 5.6% (95% CI: 2.5–11.6%) in Furnas da Boa Sorte, an isolated community of descendants of Afro-Brazilian slaves in Mato Grosso do Sul [22].

The geographic distribution of HTLV-1 infection has been shown to be heterogeneous [44]. Among the eight communities studied, the circulation of HTLV-1 was confirmed only in São José do Icatú, corroborating our previous reports suggesting that the “founder effect” phenomenon is a determinant of these differences in the geographic distribution of the virus [33,34].

The molecular and phylogenetic characterization of HTLV-1 circulating in São José de Icatú revealed the presence of the cosmopolitan subtype, transcontinental subtype (HTLV-1aA), which has been described as the most frequent subtype in populations of Pará [33,45,46], and its entry into the country was associated with the slave trade during the colonial period [31].

In our study, we investigated the occurrence of a familial cluster of HTLV-1 transmission and observed that the prevalence rate of HTLV-1 among family members of index cases was 9.52%, lower than that described in other studies in Brazil, where the prevalence of the intrafamily ranged from 22% to 64% [18,19,20,22,23,47], which revealed that vertical and sexual routes were the main transmission routes in the family clusters investigated. The genetic similarity observed between the sequenced samples, as well as the interrelationship of individuals, reinforce the theory of sexual transmission of HTLV-1 in the community.

Analysis of positive cases based on interviews revealed that the three HTLV-1-positive women (ICA #005, ICA #076, and IC #083) had a sexual relationship with the male index case (ICA #039). It is important to highlight that although index case ICA #005 reported having had four other relationships in the past, two of them tested negative for the infection, and the other two could not be tested due to one being deceased and the other not being found.

It was not possible to establish a possible chronology for this case of HTLV transmission in the Icatú community, based on the reports obtained in the interviews. However, it is possible to infer that the man (ICA #039) acquired the infection by sexual intercourse, as he reported having had sex in exchange for money, or through a blood transfusion in 2017 and then transmitted it to his partners, as there were reports of nonuse/irregular use of condoms by these individuals, a behavior that raises the risk of sexual transmission, in addition to transmission being most efficient through sexual contact from men to women [5,48,49].

The risk of spreading the infection within the community exists to the extent that nonuse and/or occasional use of condoms during sexual relations were reported by more than half of the individuals, similar to another study of *quilombola* populations in the state of Pará, in which 56.7% said they did not use condoms [34]. These results highlight the need to disseminate knowledge about measures to prevent HTLV transmission in vulnerable communities with limited access to public health services.

Among the four cases of HTLV-1-seropositive individuals detected in the present study, proviral DNA could not be detected using qPCR, which may be due to the low proviral load generally found in individuals who acquired the infection by sexual transmission [5,50,51,52]. In this case, two blood collections followed by DNA extraction were carried out at two different times, and in both, the molecular tests were negative for HTLV-1 and HTLV-2, and only the endogenous control gene (albumin) was amplified, thus ruling out interference due to the low quality of the extracted DNA. For this sample, confirmation of HTLV-1 infection was only possible through Western blot analysis, which corroborates the recommendation of Costa et al. [50] for the use of this serological confirmatory method for negative samples in qPCR, reinforcing the importance of carrying out complementary tests to confirm and differentiate HTLV-1/2 infection in a more reliable way.

Three of our four positive patients were women, were all black, and were aged older than 50 years. In Brazil, HTLV-1 is more prevalent in females, black or brown people, people with low education levels, and people in older age groups [33,53,54,55]. This epidemiological profile might represent the greatest vulnerability to which *quilombola* women are exposed as a result of the low education and sex inequalities present in *quilombola* communities, where there is a lack of adequate public education. The habit of young women having relationships with older men increases the risk of exposure to STIs, as does the lack of adequate information about prevention methods [56].

The greater predominance of women can also be attributed to their greater interest in undergoing exams/seeking medical care during expeditions to the studied communities. A greater presence of women during sample collection was clearly observed in comparison to men. Another explanation could also be that the healthcare encounters in which the samples were obtained took place primarily on weekdays when most of the men were at work and unable to attend the services, while most of the women carried out domestic activities or functions in the community and were more accessible to attend the visits. 

Our study presents some limitations, as the inability to detect proviral DNA in some cases may be attributed to the low proviral load, particularly in sexually transmitted cases. It could affect the ability of the study to fully assess the extent of HTLV-1 transmission in the community. Furthermore, the self-reported data, such as sexual behavior and condom use, also could introduce reporting bias and affect the accuracy of the results.

Finally, it is important to highlight that São José do Icatú is a rural community of *quilombos* remnants founded in approximately 1770 by black people who fled from farms in the region and who had contact with surrounding indigenous groups. This community grew over time, not only through the addition of newly escaped slaves, but also, after the abolition of slavery in Brazil, through contact with other freed slaves and rubber tappers [57]. Currently, the Icatú community is composed of 82 families, in which there is a great degree of consanguinity, and much of the population makes a living from rural activities such as agriculture [58]. The population has difficulty accessing public health services, no different from other *quilombola* communities in the state of Pará [39,59,60,61]. During our investigation and provision of healthcare through physician visits and laboratory exams, it was possible to obtain reports from individuals, including elderly individuals, who declared that they had never undergone any type of exam, which highlights the fragility and lack of public health services in this community. Therefore, the provision of health services that enable the early diagnosis of silently transmitted infectious diseases such as HTLV-1 will be key to the prevention and spread of these viruses.

## 5. Conclusions

HTLV-1 is silently transmitted in the community of São José de Icatú between individuals who have or have had some type of familial relationship, which makes it essential to implement educational programs to understand HTLV and methods of infection prevention and control. To decrease the likelihood of severe clinical impacts of the various diseases associated with HTLV-1, it is also important to prepare and establish a program for the counseling and monitoring of confirmed cases of infection in order to detect the first signs and symptoms of HTLV-1-associated diseases.

## Figures and Tables

**Table 1:** Serological and molecular characterization of HTLV-1/2 infection among the communities investigated.

Communities	N	ELISA n (%)	qPCR		Western Blot	
			HTLV-1 n (%)	HTLV-2 n (%)	HTLV-1 n (%)	HTLV-2 n (%)
Itacoã-Miri	31	0	-	-	-	-
Jacarequara	34	0	-	-	-	-
Baiquara	13	0	-	-	-	-
Bom Remédio	34	0	-	-	-	-
Caeté	13	0	-	-	-	-
Ramal do Piratuba	26	0	-	-	-	-
São José do Icatú	64	2 (3.12)	1 (1.56)	-	2 (3.12)	-
Tambaiaçú	95	0	-	-	-	-
Total	310	2 (0.64)	1 (0.32)	-	2 (0.64)	-

**Table 2:** Sociodemographic profile of individuals participating in the study.

Sociodemographic Variable	n = 310	%
**Sex**		
Female	232	74.8
Male	78	25.2
**Age**		
07 a 11	13	4.2
12 a 18	38	12.3
19 a 29	61	19.7
30 a 59	138	44.5
≥60	58	18.7
Noi reported	2	0.6
**Skin color**		
Black	229	73.9
Brown	69	22.3
Yellow	2	0.6
White	6	1.9
Not reported	4	1.3
**Marital status**		
Married/Stable union	167	53.9
Divorced	8	2.6
Single	125	40.3
Widower	8	2.6
Not reported	2	0.6
**Education**		
Illiterate	5	1.6
Literacy	154	49.7
Elementary school	74	23.9
Complete high school	70	22.6
University education	6	1.9
Not reported	1	0.3
**Income**		
<1	216	69.7
1–2	76	24.5
≥3	1	0.3
Not reported	17	5.5

**Table 3:** Risk factors for HTLV-1/2 infection evaluated in the studied populations.

Risk Factor	n = 310	%
**Tattoo**		
Yes	42	13.5
No	267	86.1
Not reported	1	0.3
**Piercing**		
Yes	7	2.3
No	303	97.7
**Illicit Drug Use**		
Yes	17	5.5
No	291	93.9
Not reported	2	0.6
**Blood transfusion**		
Yes	18	5.8
No	292	94.2
**Breastfed as a child**		
Yes	290	93.5
No	11	3.5
Not reported	9	2.9
**Sexually Active**		
Yes	217	70.0
No	76	24.5
Not reported	4	1.3
**Use of Condoms**		
Yes	67	21.6
Sometimes	51	16.5
No	136	43.9
Not reported	56	18.1
**Sexual partners per week**		
1	218	70.3
≥2	4	1.3
Not reported	88	28.4
**Having sex for money**		
Yes	9	2.9
No	272	87.7
Not reported	29	9.4
**STI history**		
Yes	8	2.6
No	265	85.5
Unknown	35	11.3
Not reported	2	0.6

**Table 4:** Common polymorphisms in the HTLV-1 LTR sequence, considering 2742LE A as the reference strain.

Sample	Nucleotide Position													
	123	125	155	157	190	203	273	398	510	545	569	570	779	123
2472LE A *	G	A	G	A	C	T	G	C	C	T	T	C	C	G
ICA #083	A	G	C	G	T	C	A	T	T	C	C	T	T	A
ICA #039	A	G	C	G	T	C	A	T	T	C	C	T	T	A
ICA #076	A	G	C	G	T	C	A	T	T	C	C	T	T	A

* Reference strain.

## Data Availability

The data analyzed in this study are included within the paper.
